# Impact of a quality improvement initiative with a dedicated anesthesia team on outcomes after surgery for adult congenital heart disease

**DOI:** 10.1016/j.xjon.2023.04.016

**Published:** 2023-05-02

**Authors:** Bill Walsh, Brigitte Mueller, S. Lucy Roche, Rafael Alonso-Gonzalez, Emily Somerset, Minako Sano, Milca Villagran Schmidt, Edward Hickey, David Barron, Jane Heggie

**Affiliations:** a Department of Anesthesia and Pain Medicine, Peter Munk Cardiac Centre, Toronto General Hospital, Toronto, Ontario, Canada; b Ted Rogers Computational Program, Peter Munk Cardiac Centre, Toronto General Hospital, Toronto, Ontario, Canada; c Division of Cardiology, Toronto Congenital Cardiac Centre for Adults, Peter Munk Cardiac Centre, Toronto General Hospital, Toronto, Ontario, Canada; d Division of Cardiac Surgery, Peter Munk Cardiac Centre, Toronto General Hospital, Toronto, Ontario, Canada; e Division of Cardiac Surgery, Hospital for Sick Children, Toronto, Ontario, Canada

**Keywords:** adult congenital heart disease, blood transfusion, cardiac surgery, morbidity, mortality, quality improvement, risk prediction

## Abstract

**Objectives:**

A quality improvement initiative was introduced to the adult congenital cardiac surgery program at Toronto General Hospital in January 2016. A dedicated Adult Congenital Anesthesia and intensive care unit team was introduced within the cardiac group. The use of factor concentrates was introduced. The study compares perioperative mortality, adverse events, and transfusion burden before and after this process change.

**Methods:**

We performed a retrospective analysis of all adult congenital cardiac surgeries from January 2004 to July 2019. Two groups were analyzed: patients undergoing operation before and after 2016. The primary outcome was in-hospital mortality. One-year mortality and prevalence of key morbidities were analyzed as secondary outcomes. A separate analysis looked at patients who had and had not attended an anesthesia-led preassessment clinic.

**Results:**

In-hospital mortality was significantly reduced in patients undergoing operation after 2016 (1.1% vs 4.3%, *P* = .003) despite a higher risk profile. One-year mortality (1.3% vs 5.8%, *P* = .003) and ventilation times (5.5 hours [3.4-13.0] vs 6.3 hours [4.2-16.2], *P* = .001) were also reduced. The incidence of stroke and renal failure was similar between groups. Blood product exposure was comparable, but the incidence of chest reopening decreased (1.8% vs 4.8%, *P* = .022), despite more patients with multiple previous chest wall incisions, on anticoagulation, and with more complex cardiac anatomy. There were no significant outcome differences between those who did or did not attend the preassessment clinic.

**Conclusions:**

Both in-hospital and 1-year mortality were significantly reduced after the introduction of a quality improvement program, despite a higher risk profile. Blood product exposure remained unchanged, but there were less chest reopenings.

## Graphical abstract

**Figure:**
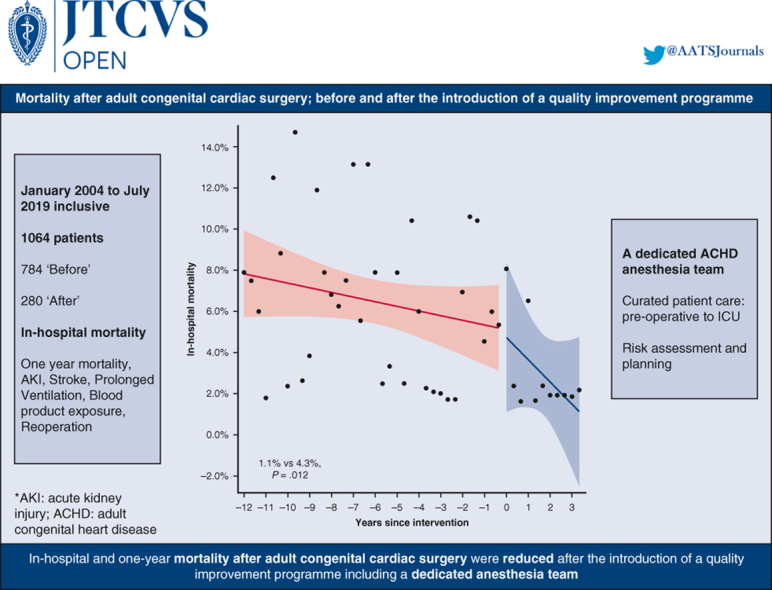

**Figure:**
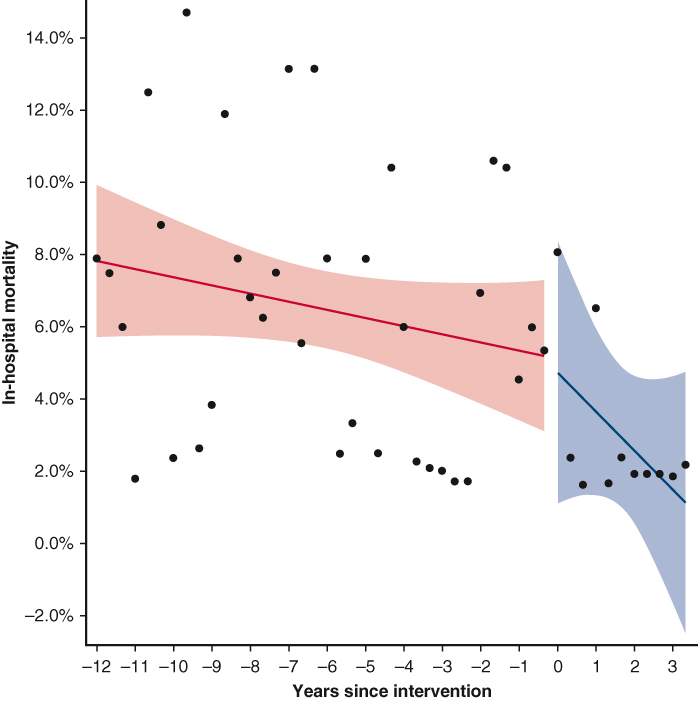
Monthly in-hospital mortality rates before and after introduction of the program changes.

**Central Message**

In-hospital and 1-year mortality decreased after adult congenital cardiac surgery at Toronto General after the introduction of a quality improvement program despite patients with a higher risk profile.

**Perspective**

Adult patients with congenital heart disease have comorbidities than can predict outcomes after surgery. Knowledge of these risk factors and management by dedicated expert teams could improve planning and perioperative and postoperative care in adult congenital cardiac surgery, and potentially lead to improved outcomes.

The population of adults living with congenital heart disease is increasing.^1^ More than 85% of patients with congenital heart disease now live into adulthood.^2^ Mortality after cardiac surgery in patients with adult congenital heart disease (ACHD) ranges from 0.7% to 9.4%.^2^, ^3^, ^4^, ^5^, ^6^

Risk prediction tools currently applied in this population are generally those originally described for pediatric cardiac surgery. These scoring systems have been shown to poorly predict mortality and adverse outcomes in adults.^3^^,^^4^^,^^7^ Patient-specific variables considered in these scores such as low weight or prematurity do not account for risk in ACHD.^8^ Procedure-specific risk estimation is based on an aggregate of all age groups.^3^ These scores lacked discriminative power when externally validated in an adult population.^8^ The Adult Congenital Heart Surgery score, an adult congenital heart specific score derived from the Society of Thoracic Surgeons Congenital Heart Surgery Database, has performed variably, likely because it is based purely on the procedure undertaken and ignores patient-specific factors.^3^^,^^9^^,^^10^

A retrospective study performed at our institution used multivariate regression to identify risk factors associated with poor outcomes after cardiac surgery in this population.^6^ Mayo End-Stage Liver Disease modified score (MELD-xi) (odds ratio [OR], 1.38, 95% confidence interval [CI], 1.23-1.54, *P* < .001), presence of cognitive impairment (OR, 3.55, 95% CI, 1.38-9.12, *P* = .009), more than 3 previous chest wall incisions (OR, 2.22, 95% CI, 1.02-4.85, *P* = .045), and anatomy other than a biventricular subaortic left ventricle (other anatomy) (OR, 3.81, 95% CI, 1.79-8.11, *P* = .001) were found to be statistically associated with poor outcomes (a composite outcome of 30-day mortality, acute kidney injury [AKI] requiring renal replacement therapy [RRT], or prolonged ventilation of more than 7 days). This has gone some way to addressing the lack of risk prediction presently available for patients with ACHD undergoing cardiac surgery.

This improved understanding of risk factors prompted the introduction of a quality improvement program with a focus on perioperative care at our institution. In January 2016, the adult congenital cardiac surgery program instituted a suite of program changes in an attempt to improve outcomes in patients identified as being at higher risk. A dedicated ACHD anesthesia team was introduced within the cardiac group. This group was responsible for the patient's entire perioperative journey through the preassessment clinic, intraoperative care, and intensive care unit stay. We hypothesized that better risk prediction at the preassessment clinic would lead to lower surgical mortality and morbidity. At the same time, the use of factor concentrates was also introduced to the surgical program, specifically prothrombin complex concentrate (PCC) and fibrinogen concentrate.

The aim of the present study was to describe mortality, adverse perioperative events, and transfusion burden before and after these program changes implemented in January 2016. We also aimed to specifically analyze the impact of attendance at the preassessment clinic on mortality and adverse surgical outcomes.

## Material and Methods

The Research Ethics Board of University Health Network approved the study protocol and publication of data (Research Ethics Board 15-9178, July 3, 2015, renewed July 3, 2021). Patient written consent for the publication of the study data was waived by the Research Ethics Board due to the retrospective and anonymous nature of the study. After obtaining this approval, a retrospective analysis was conducted of all cardiac surgeries performed in a single center in patients with congenital heart disease more than 16 years from January 1, 2004, to July 31, 2019. The following operations were excluded from analysis: heart transplant, lung transplant, transvenous or epicardial pacemaker implantation or revision, chest reexploration for bleeding or tamponade, and reexploration for a revision of the initial repair during the same admission. The setting was a quaternary referral ACHD cardiac surgery program located within a quaternary adult cardiac surgery program, where ACHD cardiac surgeries are performed by experienced congenital heart disease surgeons with an exclusive congenital heart disease practice. Data were analyzed in 2 cohorts, before and after January 1, 2016.

### Adult Congenital Heart Disease Cardiac Surgery Quality Improvement Initiatives

Initiative 1 was the creation of a dedicated subspecialty ACHD cardiac anesthesia and intensive care unit team: Before January 1, 2016, care for patients with ACHD undergoing cardiac surgery could be provided by any member of a large group of cardiac anesthesiologists (n = 40) and intensivists (n = 10) within the wider cardiac surgery program. Creation of a dedicated subspeciality ACHD cardiac anesthesia team was introduced into clinical practice on January 1, 2016. After that date, members of the ACHD cardiac anesthesia team (n = 3) coordinated care for all ACHD patients undergoing cardiac surgery, from preoperative assessment through intraoperative care, until stable postoperative management was established in the cardiac surgical intensive care unit.

Initiative 2 was the introduction of a preoperative ACHD anesthesia assessment clinic: After January 1, 2016, the ACHD cardiac anesthesia team aimed to see all patients with ACHD before cardiac surgery in a new, dedicated ACHD preoperative anesthesia clinic. At the clinic, individual risk assessment is performed using previously identified risk factors (cognitive impairment, MELD-xi score, more than 3 previous chest wall incisions, and anatomy other than a subaortic systemic left ventricle)^6^ and advance care planning conversations take place to discuss potential adverse outcomes of surgery, identify substitute decision makers, and clarify goals of care. These clinic assessments were relayed to the ACHD team at multidisciplinary case conference discussions to inform and tailor the operative plan.

Initiative 3 was the optimization of perioperative coagulation. During the same time frame use of factor concentrates, PCC and fibrinogen concentrate were introduced to the institution's cardiac surgical program targeting normalization of clotting profile in the operating room.

### Data Collection and Analysis

Patients with ACHD cardiac surgery were identified from the prospectively maintained cardiac surgery database. Patient's electronic healthcare records were accessed for data collection. The patients were divided into 2 groups for analysis: all those who underwent surgery from January 1, 2004, to December 31, 2015, inclusive (the “before” group) and those who underwent surgery from January 1, 2016, to July 31, 2019 (the “after” group). Twenty-three preoperative variables were collected for each patient (Table 1). Anatomy was defined as a 2 ventricle, subaortic left ventricle or other. Chest wall incisions included thoracotomy or sternotomy. The primary outcome was in-hospital mortality in each group. The secondary outcomes were 1-year mortality, duration of postoperative mechanical ventilation, incidence of AKI requiring institution of RRT, incidence of stroke (defined as a new-onset neurological insult as diagnosed by a neurologist or stroke physician), incidence of intraoperative blood product exposure (BPE) (a dichotomous outcome defined as the exposure to any of packed red blood cells, plasma, platelets, or cryopreciptitate), and incidence of chest reopening for bleeding or tamponade. A composite outcome of in-hospital mortality, prolonged mechanical ventilation for greater than 7 days, or AKI requiring RRT was also compared. The same preoperative variables and outcomes were also compared between those who received blood products and those who did not.

**Table 1:** Baseline characteristics and demographics

Variables	n	Overall n = 1064 Stat	n	2004-2015 n = 784 Stat	n	2016-2019 n = 280 Stat	*P* value
Anatomy (others vs 2 ventricle subaortic LV)	1055	136 (12.9%)	784	76 (9.7%)	271	60 (22.1%)	<.001
No. of chest wall incisions	1051		784		267		<.001
0		372 (35.4%)		306 (39.0%)		66 (24.7%)	
1		305 (29.0%)		242 (30.9%)		63 (23.6%)	
2		207 (19.7%)		154 (19.6%)		53 (19.9%)	
3		86 (8.2%)		57 (7.3%)		29 (10.9%)	
4		44 (4.2%)		19 (2.4%)		25 (9.4%)	
5		17 (1.6%)		5 (0.6%)		12 (4.5%)	
6		8 (0.8%)		1 (0.1%)		7 (2.6%)	
7		6 (0.6%)		0 (0.0%)		6 (2.2%)	
8		4 (0.4%)		0 (0.0%)		4 (1.5%)	
9		1 (0.1%)		0 (0.0%)		1 (0.4%)	
11		1 (0.1%)		0 (0.0%)		1 (0.4%)	
Age	1064	35 (26-47)	784	35 (25-46)	280	37 (27-48)	.087
Diabetes mellitus	1039	38 (3.7%)	769	27 (3.5%)	270	11 (4.1%)	.71
Cognitive impairment	1052	57 (5.4%)	782	44 (5.6%)	270	13 (4.8%)	.76
Atrial arrhythmia	1051	250 (23.8%)	781	191 (24.5%)	270	59 (21.9%)	.41
Anticoagulation	1053	186 (17.7%)	783	123 (15.7%)	270	63 (23.3%)	.006
Down syndrome	1048	28 (2.7%)	779	22 (2.8%)	269	6 (2.2%)	.83
Cyanosis	1054	28 (2.7%)	784	24 (3.1%)	270	4 (1.5%)	.193
FVC	523	79 (67-88)	313	78 (67-88)	210	80 (68-89)	.33
Bilirubin [mg/dL]	840	0.64 (0.47-0.94)	593	0.64 (0.41-0.88)	247	0.70 (0.53-1.08)	<.001
Albumin [g/dL]	704	42 (40-44)	523	42 (40-44)	181	42 (39-45)	.64
INR	1053	1.04 (0.99-1.14)	783	1.05 (0.98-1.14)	270	1.03 (1.00-1.12)	.59
Creatinine [mg/dL]	1053	0.84 (0.75-0.97)	784	0.84 (0.74-0.97)	269	0.87 (0.78-1.00)	.003
MELD-XI	831	7 (6-9)	585	7 (6-9)	246	7 (6-9)	.34
Systemic EF	935		781		154		.92
>55%		697 (74.5%)		579 (74.1%)		118 (76.6%)	
40%-55%		184 (19.7%)		155 (19.8%)		29 (18.8%)	
25%-39%		49 (5.2%)		42 (5.4%)		7 (4.5%)	
<25%		5 (0.5%)		5 (0.6%)		0 (0.0%)	
Weight	270	73.0 (62.0-84.0)	0	---	270	73.0 (62.0-84.0)	---
Height	270	1.69 (1.60-1.76)	0	---	270	1.69 (1.60-1.76)	---
BMI	1032	25.1 (21.8-29.0)	762	25.1 (21.9-29.0)	270	25.39 (21.72-29.17)	.97
Gender	1064		784		280		.051
Female		491 (46.1%)		376 (48.0%)	NA	115 (41.1%)	
Male		573 (53.9%)		408 (52.0%)	NA	165 (58.9%)	
Octaplex use	280	55 (19.6%)	0	0	280	55 (19.6%)	1
Fibrinogen use	280	61 (21.8%)	0	0	280	61 (21.8%)	1
FEV1	528		318		210		1
<40		18 (3.4%)		11 (3.5%)	NA	7 (3.3%)	
40+		510 (96.6%)		307 (96.5%)	NA	203 (96.7%)	

Dichotomous variables are displayed as frequency (percentage). Continuous variables are displayed as median (interquartile range). *LV*, Left ventricle; *FVC*, forced vital capacity; *INR*, International Normalized Ratio; *MELD-XI*, Model for End-Stage Liver Disease modified score; *EF*, ejection fraction; *BMI*, body mass index; *FEV1*, forced expiratory volume in 1 second.

Further analysis of the “after” group was also performed to attempt to delineate any association of the introduction of the specialist ACHD anesthesia preoperative clinic on the composite outcome of in-hospital mortality, prolonged ventilation for more than 7 days, or AKI requiring RRT. Heart transplant recipients were included in this analysis because the ACHD surgical team assumed care of ACHD transplants toward the end of 2015. Propensity score matching (1:1 matching) was used to allow the effects of treatments to be estimated on the observations by reducing the potential treatment selection bias.^11^ Preoperative clinic attenders (intervention group) were matched to nonattenders using similar preoperative variables (Tables E1 and E2). The McNemar test for matched pairs was used to compare groups.

#### Statistical methods

Clinical characteristics were summarized using descriptive statistics. Continuous variables were characterized using median and interquartile range; dichotomous or polytomous variables were characterized using frequencies. Between-group comparisons were evaluated using Wilcoxon rank-sum tests for continuous variables and Fisher exact tests for dichotomous and polytomous variables.

The monthly morbidity rate/mortality time series surrounding the introduction of the program changes was analyzed using an interrupted time series model. Each year was divided into 3 equal parts: January to April, May to August, and September to December. Within each time interval, outcome rates were computed. Percentages were subjected to empirical logistic transformation.

## Results

A total of 1064 operations performed in 1032 patients were included in the study. There were 784 patients in the “before” group and 280 patients in the “after” group. The baseline characteristics of the groups are displayed in Table 1. There was a significantly higher incidence of preoperative risk factors in the “after” group. The number of patients with other anatomy, with more than 3 previous chest wall incisions, and on preoperative anticoagulation, as well as bilirubin and creatinine levels, were all significantly higher. Sixty patients (22.1%) in the “after” group had other anatomy compared with 76 patients (9.7%) in the “before” group (*P* < .001). Table 2 describes expanded diagnostic categories for both groups. Eighty-five patients (31.9%) in the “after” group had 3 or more prior chest wall incisions compared with 82 (10.4%) in the “before” group (*P* < .001). Sixty-three patients (23.3%) in the “after” group were anticoagulated before surgery compared with 123 (15.7%) in the “before” group (*P* = .006). The median bilirubin level in the “after” group was 0.70 mg/dL (0.53-1.08) compared with 0.64 mg/dL (0.41-0.88) in the “before” group (*P* < .001), and the median creatinine level in the “after” group was 0.87 mg/dL (0.78-1.00) compared with 0.84 mg/dL (0.74-0.97) in the “before” group (*P* < .003).

**Table 2:** Diagnostic categories

Anatomy	2004-2015 n = 784	2016-2019 n = 280	*P* value
Ebstein	20 (2.6%)	10 (3.6%)	.401
Fontan	17 (2.2%)	18 (6.4%)	.002
Pulmonary atresia	3 (0.4%)	7 (2.5%)	.0045
Atrial switch D-TGA	3 (0.4%)	2 (0.7%)	.612
Arterial switch TGA	11 (1.4%)	4 (1.4%)	1
Rastelli	8 (1%)	10 (3.6%)	.011
Eisenmenger	1 (0.1%)	1 (0.4%)	.457
Cyanotic non-Eisenmenger	14 (1.8%)	3 (1.1%)	.581
Bicuspid aortic valve	54 (6.9%)	26 (9.3%)	.189
AVSD	23 (2.9%)	21 (7.5%)	.002
Coarctation	29 (3.7%)	6 (2.1%)	.246
ASD septal PAPVD	169 (21.6%)	24 (8.6%)	<.0001
VSD	34 (4.3%)	7 (2.5%)	.207
TOF/conotruncal/2 ventricle	283 (36.1%)	101 (36%)	1
ccTGA	21 (2.7%)	9 (3.2%)	.675
Others	94 (12%)	31 (11.1%)	.746

Variables are displayed as frequency (percentage). *TGA*, Transposition of the great arteries; *AVSD*, atrioventricular septal defect; *ASD*, atrial septal defect; *PAPVD*, partial anomalous pulmonary venous drainage; *VSD*, ventricular septal defect; *TOF*, tetralogy of Fallot.

**Figure 1:**
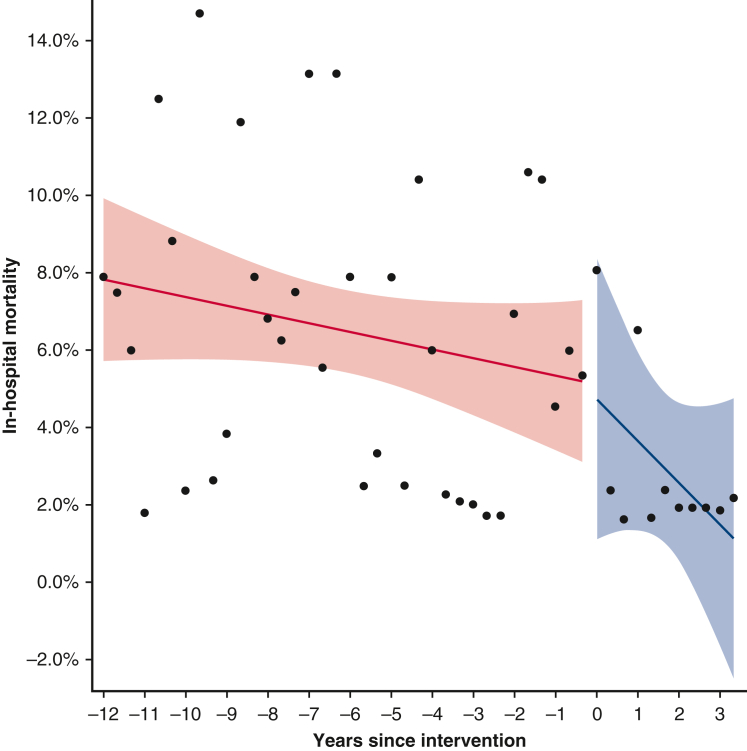
Interrupted time series model for in-hospital mortality. The monthly in-hospital mortality rate surrounding the program changes was analyzed using an interrupted time series model. Each year was divided into 3 equal parts: January to April, May to August, and September to December. Within each time interval, outcome rates were computed. Percentages were subjected to empirical logistic transformation. Before the program changes, in-hospital mortality rates were decreasing at a yearly rate of −0.075% (95% CI, –0.179 to 0.028, *P* = .154). At the time of the intervention, there is a decrease in in-hospital mortality rate of −0.091% (95% CI, –4.764 to 4.583, *P* = .97). The sustained effect is a decrease in the yearly rate of change of the in-hospital mortality rate of −0.285% (95% CI, –0.908 to 0.339, *P* = .37) after the intervention. The analysis suggests that after the program changes, the in-hospital mortality rate decreased at a yearly rate of 0.36%.

**Figure 2:**
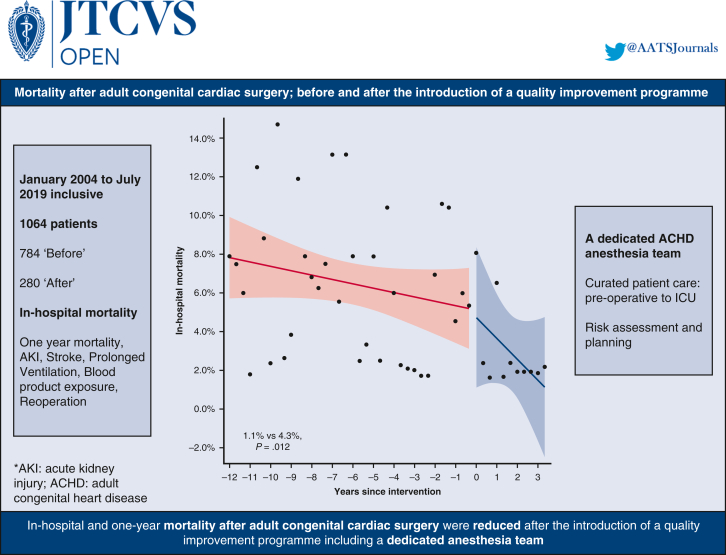
Mortality after adult congenital cardiac surgery, before and after the introduction of a quality improvement program, was assessed. A dedicated ACHD anesthesia team was introduced within the cardiac group. Use of factor concentrates was introduced contemporaneously. In-hospital and 1-year mortality rates were reduced. Incidence of AKI, stroke, and BPE was unchanged. Chest reexplorations were reduced. This was despite more complex patients. *AKI*, Acute kidney injury; *ICU*, intensive care unit; *ACHD*, adult congenital heart disease.

In-hospital mortality was 1.1% (3/274) in the “after” group compared with 4.3% in the “before” group (34/784) (*P* = .012) (Table 3). The interrupted time series model (Figures 1 and 2) shows that before the program changes the in-hospital mortality rate was decreasing at a yearly rate of 0.075% from January 2004 to December 2015. From January 2016 to July 2019, this decreased at a yearly rate of 0.36%.

**Table 3:** Outcomes

Variables	N	Overall n = 1064 Stat	n	2004-2015 n = 784 Stat	n	2016-2019 n = 280 Stat	*P* value	95% CI
In-hospital mortality	1058	37 (3.5%)	784	34 (4.3%)	274	3 (1.1%)	.012	
Ventilation time (h)	1064	6.250 (4.00-15.27)	784	6.383 (4.17-16.17)	280	5.50 (3.42-13.00)	.001	−1.58 to −0.410
Postrenal failure	1054	33 (3.1%)	784	26 (3.3%)	270	7 (2.6%)	.69	
Stroke	1054	19 (1.8%)	784	15 (1.9%)	270	4 (1.5%)	.79	
Composite outcome	1054	69 (6.5%)	784	54 (6.9%)	270	15 (5.6%)	.57	
1-y mortality	1058	44 (4.16%)	657	38 (5.8%)	238	3 (1.3%)	.003	
RBC use	1064	590 (55.5%)	784	433 (55.2%)	280	157 (56.1%)	.83	
Plasma use	1064	436 (41.0%)	784	337 (43.0%)	280	99 (35.4%)	.028	
Platelets use	1064	488 (45.9%)	784	342 (43.6%)	280	146 (52.1%)	.015	
Cryoprecipitate use	774	101 (13.0%)	494	42 (8.5%)	280	59 (21.1%)	<.001	
Total BPE use	1064	672 (63.2%)	784	488 (62.2%)	280	184 (65.7%)	.31	
Chest reopening	1070	43 (4.0%)	784	38 (4.8%)	280	5 (1.8%)	.032	

Dichotomous variables are displayed as frequency (percentage). Continuous variables are displayed as median (interquartile range). BPE is taken as a dichotomous variable for exposure to any of the individual blood products used. Composite outcome: 30-d mortality, AKI requiring RRT, or prolonged ventilation of more than 7 days. *RBC*, Red blood cell; *BPE*, blood product exposure.

One-year mortality in the “after” group was 1.3% (3/238) compared with 5.8% (38/657) in the “before” group (*P* = .003). Analysis of the causes of death shows a heterogenous set of diagnoses with no discernible pattern. Procedures such as Ebstein surgery and Fontan revision feature highly in both groups, as might be expected (Table E3). Other secondary outcomes are displayed in Table 3. The median postoperative ventilation time was shorter in the “after” group (5.5 [3.42-13.00] vs 6.38 hours [4.17-16.17], *P* = .001). There was no difference in incidence of AKI requiring RRT (2.6% vs 3.3%, *P* = .69) or stroke (1.5% vs 1.8%, *P* = .79). There was also no difference in the composite outcome between the groups (5.6% vs 6.9%, *P* = .57). At 1-year follow up, 6 of 7 patients with AKI in the “after” group had recovered their renal function and were free of dialysis and all were alive.

**Figure 3:**
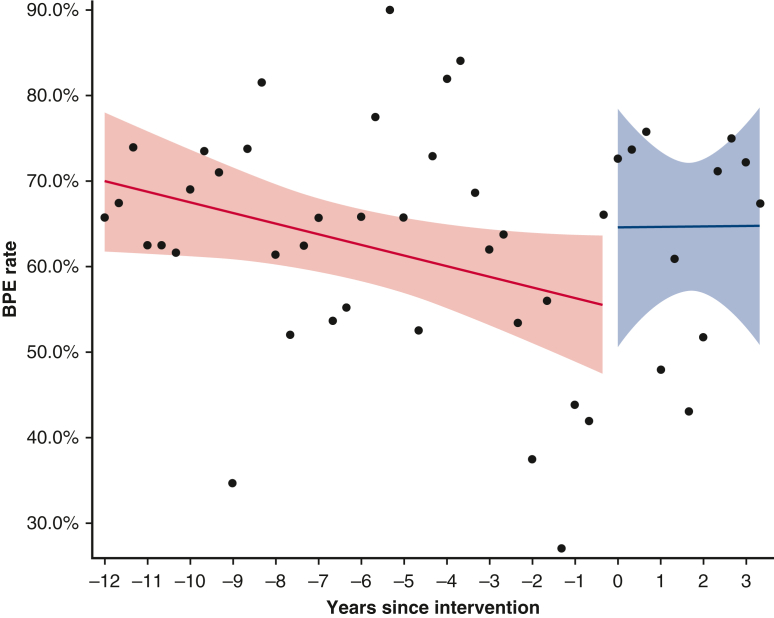
Interrupted time series model for BPE. The monthly BPE rate surrounding the program changes was analyzed using an interrupted time series model. Each year was divided into 3 equal parts: January to April, May to August, and September to December. Within each time interval, outcome rates were computed. Percentages were subjected to empirical logistic transformation. Before the program changes, BPE rates were decreasing at a yearly rate of −0.413% (95% CI, –0.811 to −0.015, *P* = .042). At the time of the intervention, there is an increase of BPE of 9.027% (95% CI, –8.963 to 27.017, *P* = .33). The sustained effect is an increase in the yearly rate of change of 0.437% (95% CI, –1.964 to 2.838, *P* = .72). The analysis suggests that after the intervention, BPE rates increase at a yearly rate 0.024%. *BPE*, Blood product exposure.

Chest reopening for bleeding or tamponade was reduced in the “after” group (1.8% vs 4.8%, *P* = .022). Incidence of BPE was also not significantly different between the groups, with 65.7% (184/280) in the “after” group and 62.2% (488/784) in the “before” group being exposed (*P* = .31). There were differences in exposure to individual blood products however. Plasma exposure was reduced in the “after” group (35.4% vs 43%, *P* = .028), whereas platelet and cryoprecipitate exposure were significantly increased (52.1% vs 43.6%, *P* = .015 and 21.1% vs 8.5%, *P* < .001, respectively). Packed red blood cell exposure was similar in both groups (56.1% vs 55.2%, *P* = .83). The interrupted time series model for BPE (Figure 3) shows that before the program changes the rate of BPE was decreasing at a yearly rate of 0.41%. At the time of the program changes BPE increased by 9.03%. After the program changes, from January 2016 to July 2019, BPE increased at a yearly rate of 0.024%.

Despite the introduction of PCC and fibrinogen, they were used in only 19.6% (55/280) and 21.8% (61/280) of patients in the “after” group respectively (Table 1). Sixty-six percent (184/280) of these patients received blood products, and 34% (96/280) did not. PCC was administered to 29.9% (55/184) of patients who received blood products compared with 0% of those who did not receive any blood products (*P* < .001). Thirty percent (56/184) of patients receiving blood products were administered fibrinogen compared with 5.2% (5/96) of those who did not receive any blood products (*P* < .001) (Table 4).

**Table 4:** Baseline characteristics and outcomes by blood product exposure

Variables	n	No BPE use n = 392 Stat	n	BPE use n = 672 Stat	*P* value
Anatomy (others vs 2 ventricle subaortic LV)	386	42 (10.9%)	669	94 (14.1%)	.153
No. of chest wall incisions	386		665		<.001
0		187 (48.4%)		185 (27.8%)	
1		112 (29.0%)		193 (29.0%)	
2		61 (15.8%)		146 (22.0%)	
3		15 (3.9%)		71 (10.7%)	
4		6 (1.6%)		38 (5.7%)	
5		3 (0.8%)		14 (2.1%)	
6		0 (0.0%)		8 (1.2%)	
7		2 (0.5%)		4 (0.6%)	
8		0 (0.0%)		4 (0.6%)	
9		0 (0.0%)		1 (0.2%)	
11		0 (0.0%)		1 (0.2%)	
Age	392	33 (25-44)	672	37 (27-49)	<.001
Diabetes mellitus	380	13 (3.4%)	659	25 (3.8%)	.86
Cognitive impairment	386	11 (2.8%)	666	46 (6.9%)	.005
Atrial arrhythmia	386	55 (14.2%)	665	195 (29.3%)	<.001
Anticoagulation	385	31 (8.1%)	668	155 (23.2%)	<.001
Down syndrome	384	8 (2.1%)	664	20 (3.0%)	.43
Cyanosis	386	7 (1.8%)	668	21 (3.1%)	.24
FVC	177	83 (70-91)	346	77 (65-87)	<.001
Bilirubin [mg/dL]	303	0.64 (0.47-0.82)	537	0.70 (0.47-0.99)	.039
Albumin [g/dL]	232	43 (41-45)	472	42 (39-44)	<.001
INR	386	1.02 (0.97-1.08)	667	1.06 (0.99-1.17)	<.001
Creatinine [mg/dL]	385	0.85 (0.77-0.97)	668	0.84 (0.75-0.98)	.49
MELD-XI	300	7 (6-8)	531	8 (7-10)	<.001
Systemic EF	353		582		.05
>55%		277 (78.5%)		420 (72.2%)	
40%-55%		57 (16.1%)		127 (21.8%)	
25%-39%		19 (5.4%)		30 (5.2%)	
<25%		0 (0.0%)		5 (0.9%)	
Weight	90	74.5 (63.0-88.2)	180	72.0 (62.0-82.2)	.062
Height	90	1.70 (1.63-1.78)	180	1.67 (1.59-1.74)	.041
BMI	377	25.4 (22.2-30.0)	655	25.0 (21.5-28.4)	.019
Gender	392		672		.048
Female		165 (42.1%)		326 (48.5%)	
Male		227 (57.9%)		346 (51.5%)	
Octaplex use	96	0 (0.0%)	184	55 (29.9%)	<.001
Fibrinogen use	96	5 (5.2%)	184	56 (30.4%)	<.001
FEV1	179		349		.135
<40		3 (1.7%)		15 (4.3%)	
40+		176 (98.3%)		334 (95.7%)	
Ventilation time (maximum set to 300 min)	392	4.333 (3.17-6.27)	672	8.500 (5.00-19.81)	<.001
Postrenal failure	386	0 (0.0%)	668	33 (4.9%)	<.001
Stroke	386	3 (0.8%)	668	16 (2.4%)	.089
In-hospital mortality	391	1 (0.3%)	667	36 (5.4%)	<.001
Composite outcome	387	2 (0.5%)	667	67 (10.0%)	<.001

Dichotomous variables are displayed as frequency (percentage). Continuous variables are displayed as median (interquartile range). *BPE*, Blood product exposure; *LV*, left ventricle; *INR*, International Normalized Ratio; *MELD-XI*, Model for End-Stage Liver Disease modified score; *EF*, ejection fraction; *BMI*, body mass index; *FEV1*, forced expiratory volume in 1 second; *FVC*, forced vital capacity.

### Impact of Blood Product Exposure on Morbidity and Mortality

A total of 672 patients (63%) received blood products (Table 4). A higher proportion of these patients were anticoagulated (23.2% vs 8.1%, *P* < .001), were older (37 years [27-49] vs 33 years [25-44], *P* < .001), and had higher prevalence of cognitive impairment (6.9% vs 2.8%, *P* = .005). They also had a higher number of chest wall incisions (21.3% had 3 or more vs 6.8%, *P* < .001), lower forced vital capacity (77% [65-87] vs 83% (70-91), *P* < .001), more atrial arrhythmias (29.3% vs 14.2%, *P* < .001), higher MELD-XI score (8 [7-10] vs 7 [6-8], *P* < .001), lower systemic ventricle ejection fraction (<55% 27.9% vs 21.5%, *P* = .05), longer ventilation times (8.5 hours [5.0-19.8] vs 4.3 hours [3.2-6.3], *P* < .001), and more postoperative AKI requiring RRT (4.9% vs 0%, *P* < .001). The in-hospital mortality was higher in patients who received blood products (5.4% vs 0.3%, *P* < .001), and a higher number of them reached the composite outcome (10.0% vs 0.5%, *P* < .001).

### Secondary Analysis

The secondary analysis included 285 surgeries on patients for a congenital lesion after January 1, 2016. A total of 234 patients attended the preoperative clinic (the intervention group), and 51 did not (the control group). Patient demographics are presented in Table E1. Thirteen patients (5.6%) in the intervention group and 6 patients (11.8%) of the control group reached the composite outcome (*P* = .12). There were 51 matched pairs. After matching the standardized mean difference (raw bias) of 33 of 37 variables decreased to less than 10% (Table E2). In the matched sample, the composite adverse outcome occurred in 9.8% of the treatment group versus 11.8% of controls (*P* = 1).

## Discussion

Mortality after cardiac surgery in adults with congenital heart disease has decreased in our center after January 2016 when compared with the preceding 12 years. This decrease is coincident with the aforementioned changes in the ACHD cardiac surgical program. Both in-hospital and 1-year mortality were reduced. Not only was the overall in-hospital mortality rate reduced in the “after” group, but its rate of decrease was greater than in the “before” group, at a rate of 0.36% per year compared with 0.075%. Ventilation times were slightly reduced, and there was no significant difference in incidence of AKI requiring RRT or stroke. There was also no difference in the composite outcome.

This was all despite more complex patients with more complex anatomy, a larger number of patients with 3 or more previous chest wall incisions, and more anticoagulated patients. Although the “after” group had statistically higher creatinine and bilirubin levels, these differences were not clinically significant.

Our results compare favorably with the published literature where mortality ranges from 0.7% to 9.4%.^2^, ^3^, ^4^, ^5^ Our contemporary cohort also compares well to reported morbidity rates with a recent similar single-center study reporting rates of AKI of 2.1%, stroke of 1.4%, and prolonged ventilation of 11.4%.^2^ Our rate of prolonged ventilation greater than 7 days was 3.9% in the “after” group.

Although overall BPE was unchanged between the 2 groups, the interrupted time series analysis shows that at the time of the program changes there was a sudden increase in BPE and that this increase was sustained. This was unexpected; however, the patients in the “after” group were more likely to be anticoagulated and have 3 or more previous chest wall incisions. Furthermore, point-of-care coagulation testing with a treatment algorithm was introduced for all cardiac surgery patients in 2015. These factors, along with the introduction of the specialist ACHD group and accompanying proactive coagulation profile management, could account for the increase. These patients received less plasma but more platelets and cryoprecipitate. This was despite the introduction of factor concentrates. Multiple small retrospective studies have shown reduced blood loss, lower red cell transfusion burden, and no increase in thromboembolic complications when using PCC after cardiopulmonary bypass compared with fresh frozen plasma.^12^, ^13^, ^14^ A meta-analysis including 14 randomized controlled trials and 1035 patients has shown that fibrinogen use in cardiac surgery reduces mortality, blood loss, and numbers of units of red blood cells when compared with placebo or other comparators.^15^ Use of factor concentrates in our study was limited. Of patients who received any blood product, only 29.9% of them were administered PCC and 30.4% fibrinogen. Of those patients who did not receive a transfusion, none of them were administered PCC and only 5.2% fibrinogen. This seems to suggest that factor concentrates were used sparingly in bleeding patients and not at all in those not being transfused. Perhaps incorporating factor concentrates into the point-of-care treatment algorithm, as reported previously, would increase use.^16^^,^^17^

Those who received blood products had a higher in-hospital mortality and incidence of AKI, and longer ventilation times. This is in line with previously published work showing increase in mortality and morbidity after transfusion in cardiac surgery.^18^^,^^19^ Whether BPE itself is the cause of increased mortality or simply a marker of more complex patients is debatable. Patients who received blood products were significantly more comorbid than those who did not.

In any case, despite overall BPE not being reduced, a proactive approach to transfusion, the coagulation profile, and intravascular volume has resulted in exposure remaining at reference levels despite more anticoagulated patients who had 3 or more previous sternotomies. Furthermore, the rate of chest reopening for bleeding or tamponade was also significantly reduced in the “after” group.

Of all the heterogenous program changes, it was thought that the introduction of the specialist ACHD team within the cardiac anesthesia group was the change most likely to impact perioperative outcomes. Although there is evidence that the impact of the anesthesiologist on perioperative mortality after cardiac surgery is negligible, the same extensive study confirms that patient risk is the single most important predictor of outcome (accounting for 95.7% of mortality variation in 10 UK centers).^20^ This group has previously established risk factors for morbidity and mortality after cardiac surgery in patients with ACHD.^6^ It was hypothesized that a preoperative assessment by the ACHD anesthesia team could be associated with a reduction in mortality due to better patient selection and decision making. Preassessment clinics have been shown to reduce duration of hospital stay in a recent systematic review.^21^ Previously used risk assessment tools in ACHD were developed for use in the pediatric population. They poorly predict mortality and morbidity in adults.^3^^,^^4^^,^^7^

To that end, a secondary analysis of the “after” group was performed. Attendance at the preoperative clinic was the intervention assessed. Although the composite outcome was achieved by only 5.6% in the intervention group compared with 11.8% in the control group, this was not significant (*P* = .12). In an effort to control for confounders, after 1:1 matching, there was no difference between the groups. There were only 51 matched pairs because only 51 patients did not attend the preassessment clinic. These were more likely to be urgent inpatients rather than elective cases. Although we cannot show that the preoperative assessment clinic alone had an impact on outcome, we believe that it does influence perioperative and postoperative management. Some benefits of the clinic, such as appreciation of previously unrecognized risk factors and delaying or postponing of surgery were difficult to measure. Furthermore, although we believe it to be an important aspect of the program changes, it is only one part of a larger heterogenous quality improvement initiative that these nonattenders still benefited from. Thus, it is difficult to demonstrate its exclusive effect.

### Study Limitations

Although mortality has decreased significantly after this initiative, we have not succeeded in pinpointing why. The retrospective nature of our study prevented us from using established quality improvement methodology. Ideally, data should be collected prospectively to document the status quo, an intervention implemented and refined, and then further prospectively collected data would document the new paradigm. Each intervention should also be studied in isolation. We believed that the most significant intervention was the establishment of a specialist ACHD anesthesia team. The preoperative clinic was an effort to better risk stratify our patients and customize their management plans. With only 51 matched pairs, we were unable to show a benefit despite the overall composite outcome being lower in the intervention group. Our study did not account for the surgical procedure. Our center is a large quaternary referral center with a high proportion of complex procedures. Procedure complexity is associated with outcome, with mortality after atrial septal defect repair being reported as low as 0.3% but that after a Fontan conversion as high as 9%.^3^^,^^4^ Cardiac transplant recipients were also excluded because the ACHD surgical team only assumed care of ACHD transplants in 2015.

## Conclusions

We performed this study to assess the impact of a suite of heterogenous quality improvement interventions on outcomes after cardiac surgery in patients with ACHD. Both in-hospital and 1-year mortality have decreased. Ventilation times have been reduced. Incidence of AKI and stroke remain unchanged. This is despite a more complex patient cohort with more complex anatomy, more chest wall incisions, and more anticoagulation. However, we have not succeeded in proving that the outcome changes were specifically related to the intervention set, thus “association” needs to be emphasized over “causation.” Our next step is to externally validate our identified patient risk factors and to build a risk prediction tool as has been done recently by a UK-based group.^9^

### Webcast

You can watch a Webcast of this AATS meeting presentation by going to: https://www.aats.org/resources/1474.

**Figure:**
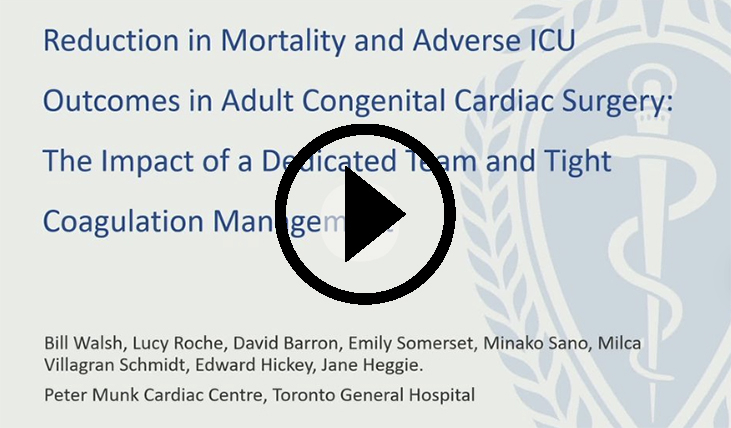

### Conflict of Interest Statement

The authors reported no conflicts of interest.

The *Journal* policy requires editors and reviewers to disclose conflicts of interest and to decline handling or reviewing manuscripts for which they may have a conflict of interest. The editors and reviewers of this article have no conflicts of interest.

## Figures and Tables

**Table E1:** Secondary analysis of effect of attendance at the preoperative anesthesia clinic: Demographics

Variable	N	Full cohort Stat	N	Nonattenders Stat	N	Clinic attenders	*P* value
Anatomy	285	122 (42.8%)	51	23 (45.1%)	234	99 (42.3%)	.76
Down syndrome	285	7 (2.5%)	51	1 (2.0%)	234	6 (2.6%)	1
No. of chest wall incisions	283	NA	51	NA	232	NA	.039
0		67 (23.7%)		6 (11.8%)		61 (26.3%)	
1		67 (23.7%)		11 (21.6%)		56 (24.1%)	
2		56 (19.8%)		11 (21.6%)		45 (19.4%)	
3		33 (11.7%)		5 (9.8%)		28 (12.1%)	
4		27 (9.5%)		6 (11.8%)		21 (9.1%)	
5		13 (4.6%)		4 (7.8%)		9 (3.9%)	
6		7 (2.5%)		2 (3.9%)		5 (2.2%)	
7		6 (2.1%)		2 (3.9%)		4 (1.7%)	
8		5 (1.8%)		3 (5.9%)		2 (0.9%)	
9		1 (0.4%)		1 (2.0%)		0 (0.0%)	
11		1 (0.4%)		0 (0.0%)		1 (0.4%)	
Previous prolonged ICU	253	23 (9.1%)	35	5 (14.3%)	218	18 (8.3%)	.34
No. of sternotomies	280		49		231		.062
0		96 (34.3%)		13 (26.5%)		83 (35.9%)	
1		107 (38.2%)		16 (32.7%)		91 (39.4%)	
2		48 (17.1%)		10 (20.4%)		38 (16.5%)	
3		17 (6.1%)		6 (12.2%)		11 (4.8%)	
4		9 (3.2%)		2 (4.1%)		7 (3.0%)	
5		2 (0.7%)		1 (2.0%)		1 (0.4%)	
6		1 (0.4%)		1 (2.0%)		0 (0.0%)	
No. of thoracotomies	275		48		227		.128
0		209 (76.0%)		32 (66.7%)		177 (78.0%)	
1		58 (21.1%)		13 (27.1%)		45 (19.8%)	
2		8 (2.9%)		3 (6.2%)		5 (2.2%)	
Age	285	37 (28-49)	51	41 (30-48)	234	36 (27-49)	.144
Emergency	281	36 (12.8%)	48	16 (33.3%)	233	20 (8.6%)	<.001
Female	285	118 (41.4%)	51	13 (25.5%)	234	105 (44.9%)	.012
Diabetes mellitus	285	13 (4.6%)	51	5 (9.8%)	234	8 (3.4%)	.062
Cognitive impairment	285	14 (4.9%)	51	1 (2.0%)	234	13 (5.6%)	.48
Atrial arrhythmia	285	68 (23.9%)	51	14 (27.5%)	234	54 (23.1%)	.59
Anticoagulation	285	79 (27.7%)	51	20 (39.2%)	234	59 (25.2%)	.057
Cyanosis	285	7 (2.5%)	51	1 (2.0%)	234	6 (2.6%)	1
FEV1	226	75 (64-85)	44	72 (61-83)	182	75 (65-86)	.45
FVC	226	79.5 (68.0-88.0)	44	80 (67-84)	182	79.0 (68.0-89.0)	.44
Bilirubin	262	12 (9-19)	49	12 (9-18)	213	12 (9-19)	.64
Albumin	195	42 (39-45)	47	41 (36-44)	148	43 (40-45)	.008
INR	285	1.03 (1.00-1.15)	51	1.10 (1.00-1.27)	234	1.03 (1.00-1.13)	.041
Creatinine	284	78 (69-89)	51	83 (72-96)	233	77 (69-88)	.015
MELD	261	7 (6-10)	49	8 (7-12)	212	7 (6-9)	.015
Systemic EF	284		50		234		.93
>55%		117 (41.2%)		19 (38.0%)		98 (41.9%)	
40%-54%		121 (42.6%)		22 (44.0%)		99 (42.3%)	
20%-39%		31 (10.9%)		6 (12.0%)		25 (10.7%)	
<20%		15 (5.3%)		3 (6.0%)		12 (5.1%)	
Weight	285	72.5 (62.0-83.7)	51	75.0 (64.5-88.2)	234	71.6 (60.2-83.0)	.065
Height	285	1.69 (1.60-1.75)	51	1.70 (1.65-1.74)	234	1.68 (1.59-1.75)	.182

Continuous variables are summarized in terms of median and interquartile range. Dichotomous and polychotomous variables are summarized in terms of the numbers and proportions of patients. *NA*, Not available; *ICU*, intensive care unit; *FEV1*, forced expiratory volume in 1 second; *FVC*, forced vital capacity; *INR*, International Normalized Ratio; *MELD*, Model for End-Stage Liver Disease; *EF*, ejection fraction.

**Table E2:** Summary of unmatched and matched variables

Variable	Unmatched data		Difference	Matched data		Difference
	No consult N = 51	Consult N = 234		No consult N = 51	Consult N = 51	
Overall distance			0.898			0.305
Simple category	0.45	0.42	−0.028	0.45	0.45	0.000
Down syndrome	0.02	0.03	0.006	0.02	0.02	0.000
0 chest wall incisions	0.12	0.26	0.143	0.12	0.06	−0.059
1 chest wall incision	0.22	0.24	0.024	0.22	0.27	0.059
2 chest wall incisions	0.22	0.19	−0.023	0.22	0.25	0.039
3+ chest wall incisions	0.45	0.30	−0.152	0.45	0.41	−0.039
Missing chest wall incisions	0.00	0.01	0.009	0.00	0.00	0.000
Previous prolonged ICU	0.10	0.08	−0.021	0.10	0.10	0.000
0 sternotomies	0.25	0.35	0.100	0.25	0.20	−0.059
1 sternotomies	0.31	0.39	0.075	0.31	0.37	0.059
2+ sternotomies	0.39	0.24	−0.149	0.39	0.37	−0.020
Missing sternotomies	0.04	0.01	−0.026	0.04	0.06	0.020
0 thoracotomies	0.63	0.76	0.129	0.63	0.67	0.039
1+ thoracotomies	0.31	0.21	−0.100	0.31	0.25	−0.059
Missing thoracotomies	0.06	0.03	−0.029	0.06	0.08	0.020
*Age*	40.73	38.24	−0.216	40.73	41.18	0.039
Emergency	0.31	0.09	−0.228	0.31	0.27	−0.039
Female	0.25	0.45	0.194	0.25	0.33	0.078
Diabetes mellitus	0.10	0.03	−0.064	0.10	0.12	0.020
Cognitive impairment	0.02	0.06	0.036	0.02	0.02	0.000
Atrial arrhythmia	0.27	0.23	−0.044	0.27	0.29	0.020
Anticoagulation	0.39	0.25	−0.140	0.39	0.35	−0.039
Cyanosis	0.02	0.03	0.006	0.02	0.02	0.000
*FEV1*	71.62	73.95	0.150	71.62	71.19	−0.028
*FVC*	76.48	78.22	0.120	76.48	77.37	0.062
*Bilirubin*	**15.38**	**15.80**	**0.037**	**15.38**	**14.05**	**−0.116**
*Albumin*	**38.90**	**41.79**	**0.405**	**38.90**	**40.62**	**0.241**
*INR*	1.23	1.19	−0.109	1.23	1.25	0.049
*Creatinine*	**100.29**	**80.35**	**−0.308**	**100.29**	**87.57**	**−0.197**
*MELD-XI*	**10.08**	**8.73**	**−0.291**	**10.08**	**9.49**	**−0.127**
Systemic EF >55%	0.37	0.42	0.046	0.37	0.39	0.020
Systemic EF 40%-54%	0.43	0.42	−0.008	0.43	0.37	−0.059
Systemic EF 20%-39%	0.12	0.11	−0.011	0.12	0.12	0.000
Systemic EF <20%	0.06	0.05	−0.008	0.06	0.12	0.059
Systemic EF Missing	0.02	0.00	−0.020	0.02	0.00	−0.020
*Weight*	76.83	72.43	−0.290	76.83	75.83	−0.066
*Height*	1.70	1.67	−0.252	1.70	1.70	0.041

The differences are expressed as standardized mean differences for continuous variables (italic font) that were standardized by the standard deviation of the control group, and raw differences for binary variables (all other variables).

Bold text signifies that after matching the standardized mean difference of the variables remained greater than 10%.

*ICU*, Intensive care unit; *FEV1*, forced expiratory volume in 1 second; *FVC*, forced vital capacity; *INR*, International Normalized Ratio; *MELD*, Model for End-Stage Liver Disease; *EF*, ejection fraction.

**Table E3:** Causes of death

Year	Cause of death	Place of death	Days postoperatively	Procedure	Emergency	Comments
2004	Thrombosis of Fontan	CVICU	8	TCPC conversion	No	HIT
2004	Coronary artery injury	CVICU	13	ToF PVR	No	ECMO postoperatively
2004	Pneumonia/HF	CVICU	7	Mustard d-TGA PA banding	No	
2005	Dialysis	Community Hospital	60	TCPC conversion	No	Died at community hospital
2005	MOF Sepsis	CVICU	3	Prosthetic aortic valve endocarditis	Yes	
2005	MOF Sepsis	CVICU	3	Pacer wire endocarditis	Yes	
2005	CVA	Readmit TGH ICU	120+	Fontan: AI post-aortic root	No	Pulmonary hemorrhage from AVM at school
2006	Stroke	CVICU	1.5	RV to PA conduit ToF	No	
2006	Sudden death	Home	9	ToF PVR	No	Died suddenly at home 3 d post-DC. ST changes day 4 postoperatively.
2007	RV failure/hypoxia	CVICU	10	Ebstein TVR	No	
2007	MOF Sepsis	CVICU	12	Aortic stenosis AVR	No	ARDS
2007	MOF Sepsis	CVICU	22	MVR, TV repair	No	Cirrhosis, renal failure
2007	Unknown	Community Hospital	39	Subaortic stenosis	No	Letter from referring cardiologist
2008	MOF Sepsis	CVICU	43	TVR, ASD closure endocarditis	Yes	Hepatitis B and C, IVDU, endocarditis
2008	MOF Sepsis	CVICU	10	DORV, Fontan endocarditis	Yes	Endocarditis
2008	MOF	CVICU	3	Take down BT shunt, Glenn, TVR, PA plasty	Yes	Hypoxia
2009	Hyperkalemia hypoxia	CVICU	1	MVR	No	Malignant AF, hypoxia; low CO, Diff airway self-extubated
2009	MOF	CVICU	122	Central shunt	No	Cyanotic heart disease single ventricle
2009	Low CO	CVICU	2	Ebstein TVR	No	Low cardiac output
2009	Intracranial hemorrhage	CVICU	67	Pulmonary homograft, heart lung Tx	No	ECMO as a bridge to Tx (failed recognition of PH)
2009	MOF	CVICU	14	Fontan revision, Maze	No	Concomitant GI bleed
2010	Ischemic bowel	CVICU	14	PA plasty with VSD Repair	No	Pneumonia
2011	Low CO	CVICU	3	Ebsteins-TVR	No	Cardiogenic shock
2011	Coronary and vertebral dissection	CVICU	60	MVR and Bentall	No	Marfans-Prolonged vent wean
2011	Unknown	Hospice	240+	PVR and TV repair		Pulmonary stenosis, liver dysfunction
2011	Stroke anoxic brain injury	MSNICU	3	Primum ASD and Maze	No	Hemorrhagic conversion of perioperative stroke
2012	Sudden death	Home	200+	ToF, PVR	No	Post–chest reopen, discharged well
2012	Sudden death	Ref Hospital	110+	Truncus		ECMO postsurgery for failing systemic vent and AI. Paraplegia
2014	MOF Sepsis	CVICU	94	Valve-sparing root, MVR	No	Marfans-Prolonged vent wean
2014	Multiple strokes	CVICU	12	ToF, PVR	No	Previous strokes
2014	Respiratory failure	CVICU	40	PVR, failed pericardiectomy	No	Pericardial restriction, severe pulmonary restriction
2014	Anoxic brain injury-MOF	Community Hospital	120	High-risk RV to PA conduit	No	Failed airway in operating room for ETT exchange
2014	Liver failure	CVICU	33	ToF PVR and TV repair	No	Childs B cirrhosis
2014	Intracranial Hemorrhage	CVICU	15	Ebstein TVR	No	RV failure
2014	MOF-Cardiogenic shock	CVICU	14	PVR, TV repair post–arterial switch	No	Cirrhosis, renal failure
2015	Intracranial hemorrhage	CVICU	16	cc-TGA RVAD	No	RVAD for cc-TGA bridge to Tx
2015	Intracranial hemorrhage	MSICU	9	ASD unroofed coronary sinus	No	ECMO for biventricular failure, 3 chest re-explorations
2015	Stroke, ACA, and MCA septic emboli	CVICU	4	ToF PVR, MVR, TVR	Yes	Endocarditis with septic emboli
2016	MOF Massive transfusion	Operating room	0	ToF-PVR	No	Sternal reentry injury Massive transfusion
2016	Low CO	CVICU	14	Ebstein TVR, rescue BD Glenn	No	MOF Difficult vent wean
2017	MOF	CVICU	1	Fontan revision-Maze	No	Failed within 24 h

*CVICU*, Cardiovascular intensive care unit; *TCPC*, total cavopulmonary connection; *HIT*, heparin-induced thrombocytopenia; *ToF*, tetralogy of Fallot; *PVR*, pulmonary valve replacement; *ECMO*, extracorporeal membrane oxygenation; *HF*, heart failure; *d-TGA*, dextro-transposition of the great arteries; *PA*, pulmonary artery; *MOF*, multiorgan failure; *CVA*, cerebral vascular accident; *TGH*, Toronto General Hospital; *AVM*, arteriovenous malformation; *AI*, aortic incompetence; *RV*, right ventricle; *DC*, discharge; *ST*, segement; *TVR*, tricuspid valve replacement; *AVR*, aortic valve replacement; *ARDS*, adult respiratory distress syndrome; *ASD*, atrial septal defect; *IVDU*, intravenous drug user; *DORV*, double outlet right ventricle; *BT*, Blalock-Taussig; *CO*, cardiac output; *Tx*, transplant; *PH*, pulmonary hypertension; *GI*, gastrointestinal; *VSD*, ventricular septal defect; *MVR*, mitral valve replacement; *MSICU*, Medical-Surgical Intensive Care Unit; *ETT*, endotracheal tube; *RVAD*, right ventricular assist device; *cc-TGA*, congenitally corrected transposition of the great arteries; *ACA*, anterior cerebral artery; *MCA*, middle cerebral artery; *BD*, bidirectional.
